# OnabotulinumtoxinA in Migraine: A Review of the Literature and Factors Associated with Efficacy

**DOI:** 10.3390/jcm10132898

**Published:** 2021-06-29

**Authors:** Jason C. Ray, Elspeth J. Hutton, Manjit Matharu

**Affiliations:** 1 Department of Neurology, Alfred Hospital, Melbourne, VIC 3004, Australia; elspeth.hutton@monash.edu; 2 Department of Neurology, Austin Health, 145 Studley Road, Heidelberg, VIC 3084, Australia; 3 Department of Neuroscience, Monash University, Melbourne, VIC 3004, Australia; 4 Headache and Facial Pain Group, University College London (UCL) Queen Square Institute of Neurology, Queen Square, London WC1N 3BG, UK; manjit.matharu@nhs.net; 5 The National Hospital for Neurology and Neurosurgery, Queen Square, London WC1N 3BG, UK

**Keywords:** migraine, botulinum toxin, predictors of efficacy

## Abstract

The efficacy of onabotulinumtoxinA (OnaB-A) as a preventative treatment for chronic migraine, emerging fortuitously from clinical observation is now supported by class one evidence and over two decades of real-world clinical data. There is still limited ability to predict a clinically meaningful response to OnaB-A for individual patients, however. This review summarises briefly the proposed mechanism of OnaB-A in chronic migraine, the literature of predictors of clinical response, and recent developments in the field.

## 1. Introduction

Migraine is the most common disabling neurological condition, which globally in 2016 affected over one billion people, causing 45.1 million years lived with disability [1]. Migraine may be classified as either episodic or chronic; patients with episodic migraine experience fewer than 15 headache days per month while patients with chronic migraine suffer 15 or more headache days per month, of which at least eight fulfil the International Classification of Headache disorders (ICHD-3) criteria for migraine [2]. Every year, 2.2–3.1% of patients with episodic migraine progress to chronic migraine, and it is estimated that 1–4% of the population meets the criteria for chronic migraine [3]. 

The treatment of chronic migraine includes the need for effective preventative treatment in order to reduce the number of attacks and thereby burden of the disease. OnabotulinumtoxinA (OnaB-A) has emerged as an effective preventative treatment of chronic migraine for many patients; however, predicting efficacy for individual patients remains challenging. In this paper, we review the use of OnaB-A in the treatment of chronic migraine, its efficacy, predictors of response, clinical considerations, and future directions. 

## 2. A Brief History of Botulinum Toxin

Botulinum toxin is one of the deadliest substances on earth. Its discovery as a therapeutic for migraine therefore was accidental and fortuitous. Botulinum toxin has seven serotypes (A–G) and is produced by clostridium botulinum, a Gram-positive anaerobic bacterium. Biological activity occurs at approximately 1 ng (a billionth of a gram), and the human median lethal dose (LD50) for inhalation botulism is 1–3 ng/kg [4,5]. Accordingly, one tablespoon of botulinum toxin would supply the world for one year [4]. 

The first scientific description of botulism was made by Dr. Kerner during the Napoleonic war (1795–1813), who described following the ingestion of contaminated meat, vomiting, intestinal spasm, ptosis, strabismus, dysphagia leading to flaccid paralysis and respiratory failure. Dr. Kerner even hypothesised that this toxin could be used therapeutically [6]. Botulinum toxin was not isolated until late in the 19th century by Emile Pierre-Marie van Ermengem [7].

**Figure 1:**
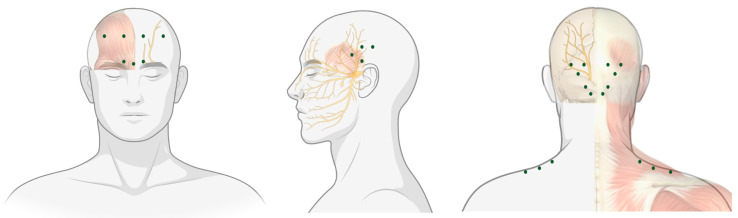
The PREEMPT protocol for injection of onabotulinumtoxinA (stylised)—green dots represent injection sites [12].

The first use of botulinum toxin therapeutically, as foreshadowed by Dr. Kerner would be by Dr. Scott in the treatment of strabismus [8]. Since then, it has been employed in the treatment of a number of conditions including muscle spasticity and hyperhidrosis. However, it was in the 1990s that Dr. Binder, a facial-plastic surgeon noted that some patients who were administered botulinum toxin for cosmesis reported an improvement in their migraine [9]. A number of open-label and observational studies with a variety of injection techniques followed, ultimately culminating in the Phase III Research Evaluating Migraine Prophylaxis Therapy (PREEMPT) study, which would provide class one evidence for the use of onabotulinumtoxinA (OnaB-A) in chronic migraine, FDA approval in 2010, and the ‘PREEMPT’ injection paradigm still used today (Figure 1) [10,11].

## 3. Mechanism of Action

**Figure 2:**
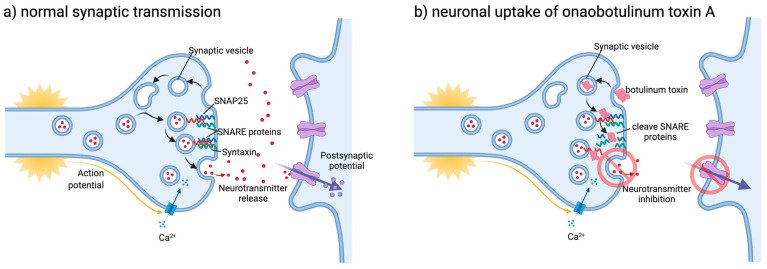
Mechanism of action of botulinum toxin [12]. (**a**) Normal synaptic transmission; arriving active channel opens voltage-gated calcium channels, allowing influx of calcium. Calcium allows synaptic vesicle docking via SNARE proteins, neurotransmitter release, and activation of post-synaptic potential. (**b**) Neuronal uptake of onabotulinumtoxinA, internalisation of botulinum toxin via synaptic vesicle, and cleavage of SNAP-25 protein causing inhibition of neurotransmitter release.

Botulinum toxin induces flaccid paralysis due to its affinity for skeletal and autonomic cholinergic nerve terminals. Botulinum toxin is internalised inside the synaptic vesicles (SV) of the presynaptic membrane. Botulinum toxins affinity to SV contribute both to its lethality and therapeutic efficacy, as hyperactive nerve terminals have a higher rate of SV endocytosis, favouring internalisation. Once internalised, botulinum toxin cleaves nine amino acids from the C-terminus of the Soluble NSF-Attachment Protein Receptor (SNARE) protein SNAP25, disrupting exocytosis and thereby inhibiting the release of acetylcholine, causing a chemical denervation and muscle relaxation/paralysis (see Figure 2) [13,14].

The mechanisms by which OnaB-A prevents chronic migraine are multifaceted and incompletely understood. Within the cranial sensory neurons, OnaB-A impairs nociception transmission by inhibiting the release of calcitonin gene related peptide (CGRP), substance P and glutamate again by cleaving SNAP25, suppressing peripheral sensitisation, with a secondary inhibitory effect on development and maintenance of central sensitisation [11,13].

Furthermore, OnaB-A interferes with ion channels including transient receptor potential cation channel vanilloid subfamily, member 1 (TRPV1), and transient receptor potential cation channel ankyrin subfamily, member 1 (TRPA1), impairing their delivery to the terminal membrane and thus downregulating receptor activity. This has a further positive impact on migraine by reducing trafficking primarily in c-type meningeal nociceptors [11].

It has also been demonstrated that OnaB-A has a direct central effect, despite the fact that it does not cross the blood–brain barrier. Through retrograde axonal transport, OnaB-A has been shown to travel to not only sensory ganglia but also to afferent innervations of the brain stem and cleave SNARE within central nervous system neurons [14].

## 4. Efficacy of OnabotulinumtoxinA

Class one evidence for botulinum toxin was established in the PREEMPT-1 and PREEMPT-2 studies. Patients aged 18–65 with chronic migraine, excluding patients with continuous headache were randomised to receive 155–195 units of OnaB-A in 31 sites over the head and neck. Between the studies, 1384 subjects were randomised to either OnaB-A or placebo. In pooled analysis, 24 weeks of OnaB-A was found to significantly reduce monthly headache days (MHD) by 8.4 compared to 6.6 with placebo, and a 50% responder rate (the proportion of the study population to achieve at least a 50% reduction in monthly migraine days) of 47.1% (placebo 35.1%) [10].

The efficacy of OnaB-A in chronic migraine has been confirmed in numerous real-world studies and subjected to several meta-analyses. In a 2018 Cochrane review of 28 randomised control trials (RCTs), OnaB-A for chronic migraine reduced the number of migraine days by 3.1 (95% CI 1.4–4.7) and headache days by 1.9 (95% CI 1.0–2.7) after six months [15]. The long-term efficacy of OnaB-A in chronic migraine was assessed in the REPOSE study, a 24-month open-label study of 641 subjects. MHD was reduced from a mean baseline of 20.5 to 7.4 after 24 months, with corresponding improvement in quality-of-life measures [16]. Three meta-analyses have not found efficacy for OnaB-A in episodic migraine [15,17,18].

The most common adverse events in the REPOSE study from OnaB-A include eyelid ptosis (5.4%), neck pain (2.8%) and musculoskeletal stiffness (2.7%). On meta-analysis, the risk ratio of any adverse event across 15 RCTs was 1.28 (95% CI 1.12–1.47) and included ptosis (RR 7.29, 95% CI 3.18–16.73), muscle weakness (RR 13.67, 95% CI 6.73–27.75), neck pain, (RR 2.98, 95% CI 2.06–4.32) and injection site pain (RR 2.10, 05% CI 1.02–4.32). 

### 4.1. Quality of Life Measures

While emphasis is placed on reducing the frequency of headache days, improvement of quality of life, the burden of disease and reduction in mental health comorbidity are critical outcomes for both patients and treating clinicians. The 2-year COMPEL study included the patient health questionnaire (PHQ-9) and generalised anxiety disorder (GAD-7) score and found, respectively, that 78% and 81.5% of patients had significant reduction in symptoms of depression and anxiety, while other studies have reported improvement from even the first cycle of treatment [19,20].

Quality of life measures of OnaB-A therapy have been assessed in multiple studies utilising several measures including the headache impact test (HIT-6), migraine disability assessment test (MIDAS) and migraine-specific quality of life questionnaire (MSQ). A clinically meaningful reduction in HIT-6 (reduction ≥5) has been reported in 30.1–59.1% of patients in trials [21,22], while MIDAS scores reduce significantly from 67.3 to 15.3 by 6 months in real-world setting [23]. Role function domains of the MSQ improve by 14.4–33 with OnaB-A [22,24].

### 4.2. Efficacy Compared to Other Treatments

Three RCTs have compared the efficacy of OnaB-A to other established preventative treatments for chronic migraine. Two RCTs of 59 and 60 participants respectively compared OnaB-A to topiramate at a daily dose of 100–200 mg. Both groups reported similar efficacy; however, OnaB-A was associated with fewer adverse events [25,26]. A third RCT of 59 patients compared OnaB-A to sodium valproate 250mg BID and reported similar efficacy between groups, with fewer adverse events in the OnaB-A group [27].

As outlined in Table 1, earlier initiation of onaB-A in the course of the disease is associated with a favourable outcome; however, practically, several jurisdictions have differing requirements before commencing onaB-A. The comparative efficacy of OnaB-A and the new CGRP monoclonal antibodies (mAb) has not been assessed in clinical trials. A retrospective US claims analysis suggested that patients treated with erenumab had fewer healthcare visits related to migraine compared to OnaB-A; however, further studies are required [28]. There is insufficient evidence to make a categorical recommendation of one therapy over the other in patients who have failed to respond to oral preventative treatment. Some experts have suggested an approach of commencing onaB-A and transitioning to CGRP mAb where response is sub-optimal [29]. There is also pre-clinical data to suggest that dual therapy may be beneficial, with OnaB-A mainly inhibiting c-fibres in the trigeminovascular system, and CGRP mAb A𝛅-fibres [30]. A protocol for a network meta-analysis comparing OnaB-A and CGRP mAb is planned and may provide some clarification on relative efficacy [31].

### 4.3. Efficacy in Hemiplegic Migraine

Hemiplegic migraine, a rare subtype of migraine with aura, has no RCT proven preventative treatment due to the rarity of the condition. The use of OnaB-A in hemiplegic migraine has been reported in two case studies and a case series of eleven patients. In the case series, 9 of the 11 patients reported a decrease in the frequency of their migraine aura [53].

### 4.4. Efficacy in Medication Overuse Headache

As seen in Table 1, Medication Overuse Headache (MOH) is a common comorbidity in patients with chronic migraine, and as such, the efficacy of OnaB-A in this population is of clinical interest [54,55]. In a RCT comparing OnaB-A to acute withdrawal of medication, OnaB-A was not superior to placebo at 12 weeks in either reduction in MMD (6.2 vs. 7.0) or MHD (26.9% vs. 20.5%) [56]. Conversely, treatment with OnaB-A appears to remain efficacious in populations with high rates of MOH [57]. In a UK study, 56.2% of patients with CM and MOH had a ≥30% reduction in MHD compared to 64.9% of patients with CM alone, further supporting its efficacy [58]. Accordingly, the European Headache Federation recommends that it is preferable to withdraw acute medication prior to the initiation of OnaB-A; however, where this is not feasible, OnaB-A may be commenced prior to or while withdrawing acute medication [59].

## 5. Factors Associated with Efficacy of Botulinum Toxin

### 5.1. Clinical Factors

A clinical biomarker of efficacy of OnaB-A in chronic migraine has remained of interest to clinicians for the past three decades, in order to allow best use of healthcare resources and plan treatment. A summary of published articles that have reported on efficacy of OnaB-A in chronic migraine is presented in Table 1. While several clinical, chemical, genetic and radiological predictors have been described in the literature, at present, there is insufficient data to guide treatment decisions or predict efficacy for an individual patient.

One of the first clinical predictors of efficacy was proposed by Jakubowski, who described three phenotypes of pain in migraine: ocular (‘like pushing a finger into my eye’), imploding (‘someone is crushing my skull’) and exploding (‘like my head is going to explode’). Jakubowski et al. reported a cohort of 63 patients with both episodic and chronic migraine, who received 100 units of OnaB-A, prior to the PREEMPT protocol. They reported that an ocular or imploding phenotype of pain was associated with response to OnaB-A, while exploding pain was not [51]. This observation was duplicated by Burstein et al. in 2009, Kim et al. in 2010 and Grogan et al. in 2013, all with mixed cohorts and a non-PREEMPT protocol [47,48,49]. Lin et al. failed to demonstrate a difference between imploding and exploding pain but again reported ocular pain to be predictive of response, while Pagola et al. found no such correlation [44,45].

The second phenotypic characteristics of interest has been unilateral pain, which was first reported by Mathew et al. to be predictive of response, along with cutaneous allodynia and pericranial muscle tenderness [50]. De Tommaso et al. also reported that less severe allodynia was a predictor of clinical response, as did Young et al., however, only on long-term follow-up [36,37]. Other studies, however, have failed to find that unilateral pain [45], allodynia [33] or neck muscle tenderness [51,52] predict response to OnaB-A.

Whether the phenotypic characteristics outlined above are predictors of clinical response, and their predictive value if so, remains unclear. Certainly, no patient should have OnaB-A withheld for lacking these characteristics. Presuming a predictive value of these features, one possible mechanism suggested is that unilateral ocular pain, in the presence of autonomic symptoms, is a marker of ongoing peripheral trigeminal activation and central sensitisation [60,61].

With interest in trigeminal activation as a marker of efficacy, response to triptans, which exert some effect in reducing CGRP release from trigeminal afferents, is also of clinical interest. Lovati et al. first published an observational study in 2018 which did demonstrate that triptan responsiveness was a predictor of efficacy in OnaB-A [40]. In an attempt to confirm these findings, a prospective study rating triptan efficacy prior to commencement of OnaB-A in 49 patients was performed, which found that triptan efficacy did not predict response to OnaB-A after three months treatment [32].

Finally, assessment of patient comorbidities appears poorly predictive of response to OnaB-A. Elevated body mass index (BMI), while a risk factor for chronification of migraine, does not appear to predict response [33,42,43,44]. A shorter duration of disease has been reported to be a predictive value in some studies [42,52], but not others [33,43]. Finally, the presence of depressive symptoms again has conflicting results [35,39,42,45].

### 5.2. Biomarkers

Supporting the prospect that trigeminal activation is associated with responsiveness to OnaB-A, two studies have found that patients with elevated levels of calcitonin gene-related peptide (CGRP) were predictive of response to OnaB-A, albeit with very different clinical ranges [33,38,43]. Pentraxin-related peptide (PTX3), a marker of endothelial dysfunction, has also been reported as a predictor of OnaB-A efficacy [38]. While interesting, testing of CGRP is not yet practical at the bedside.

### 5.3. Imaging Features

Several studies have attempted to address whether structural changes exist between responders and non-responders of OnaB-A therapy. Hubbard assessed structural and functional MRI changes in non-responders to OnaB-A compared to responders who had reverted to episodic migraine. They demonstrated that OnaB-A responders had cortical thickening in the right primary somatosensory cortex, anterior insula, left superior temporal gyrus and pars opercularis compared to non-responders [41]. Previous studies have reported reduced insular thickness in high-frequency episodic migraine and in chronic migraine compared to episodic [62,63]. As the study only evaluated patients post intervention, it is unclear whether these changes represent a differentiating feature of response or reactive structural change as responders revert to episodic migraine. As noted by the authors, further prospective study in this area would be useful.

In a second study of 62 patients with chronic migraine, iron deposition in the periaqueductal gray (PAG) was found to be associated with a negative response to OnaB-A [33]. Iron deposition has been shown to be increased in CM compared to EM and in EM compared to healthy controls, suggesting both that accumulation occurs with prolonged disease and a possible mechanism by which duration of disease is a marker of OnaB-A efficacy [64]. The mechanism by which iron deposition could affect the efficacy of OnaB-A is not known. One theory is that dysfunction of the PAG, which is involved in descending anti-nociceptive function, could taper response to the more peripheral effect of OnaB-A, or that iron deposition is a biomarker of a higher activation rate of pain-circuits and thus more refractory disease [33]. 

### 5.4. Genetic Markers

One study has assessed for 25 single nucleotide polymorphisms in 156 female patients with chronic migraine. Two polymorphisms were found to be associated with response to OnaB-A; CALCA rs3781719 (40.9% vs. 26.9%) and TRPV1 rs222749 (4.17% vs. 12.5%) [34]. Whilst not accounting for the entire population, these two genes which respectively encode CGRP and the TRPV1 receptor, a target of OnaB-A, support the mechanism of action of OnaB-A in chronic migraine.

## 6. Clinical Considerations

### 6.1. Assessment of Efficacy When Commencing Botulinum Toxin

There is general consensus amongst clinicians that a reduction in headache days by 30% to 50% is clinically meaningful and a marker of efficacy of OnaB-A, with the latter used as an outcome measure in the PREEMPT trials [65,66]. In pooled analysis of the PREEMPT studies, 49.3% of participants achieved a 50% reduction in MHD in their first cycle, while 11.3% and 10.3% of patients responded only after the second and third cycle, respectively [66].

Several studies have been undertaken to assess the cumulative efficacy of commencing OnaB-A beyond the first two cycles. In a single-centre prospective study of 56 patients, the proportion of patients with a 50% reduction in MHD doubled between cycle 2 and 5 (27 to 48%) [67]. In a retrospective cohort study of two Italian centres, response to OnaB-A at 6 months was only modestly predictive of response at 12 months (Cohen’s Kappa 0.51), and 23.8% of non-responders at 6 months were responders at 12 months [68]. The predictive value of responder status was further delineated by the Italian group in an open prospective study who found that 64.7% of patients who respond in the first three cycles have sustained response, while 23.4% of initial non-responders converted in cycle four or five [69]. On the basis of this, it is recommended to trial OnaB-A for 2–3 treatment cycles in order to assess efficacy; however, local jurisdictions have differing restrictions [59].

### 6.2. Wearing Off Effect of Botulinum Toxin

Response to OnaB-A is not uniform between 12-week injections but rather has a ‘U’ shape response with clear induction, peak-effect, and ‘wear-off’ phases [70]. Wear-off of OnaB-A is a common clinical phenomenon, presenting a challenge in management. Rates of wear-off of OnaB-A have been reported between 44–62.9% of patients, most commonly 2–4 weeks before the following injection. The driving mechanism of wear-off is not clear, with no differences in patient characteristics found to be predictive of wear-off [71,72]. The duration of biological effect of OnaB-A has been speculated, with pre-clinical evidence of reversal of neuromuscular blockade seen prior to 12 weeks and histological studies in essential blepharospasm showing resolution of fibre diameter by 12 weeks [73]. Nonetheless, the duration of clinical effect of OnaB-A seems to vary between relative short durations (dystonia) and much longer (neurogenic detrusor overactivity) [74,75].

Short-term bridging prophylactic strategies are most commonly employed to treat wear-off, including intramuscular ketorolac injections and peripheral nerve blocks [71]. Increasing the dose of OnaB-A may achieve a longer duration of response in up to 75% of patients in one study [72]. Considering the frequency of OnaB-A injection for indications other than chronic migraine, it is possible that a more frequent treatment regimen may be efficacious. In treatment of cervical dystonia, blepharospasm and cosmesis, frequency of treatment varies between 8–10 weeks [71].

### 6.3. Stopping Botulinum Toxin

The European Headache Federation recommends considering stopping OnaB-A in patients who have less than ten MHD for three months [59]. In one prospective study, 49% of a cohort of 276 patients were withdrawn from OnaB-A after meeting the predefined criteria fewer than five MHD for two consecutive 12-week cycles and a MIDAS score of <10. Over six-months of follow-up, 80% of patients had no clinical worsening or need to resume preventative treatment [76]. In a smaller case series, OnaB-A was withdrawn in 54 patients who reverted to an episodic pattern, of whom 80% remained episodic migraine after six months [76].

## 7. Emerging Concepts

### 7.1. Location of Injections

The choice of sites in the PREEMPT protocol of seven specific muscle groups aligns with the peripheral nerve distribution of the trigeminal, occipital and cervical sensory nerves and has proven efficacy in chronic migraine [77]. As understanding of the mechanism of botulinum toxin in chronic migraine has evolved, alternate injection strategies have been speculated.

Injection of the sphenopalatine ganglion (SPG) has been proposed in order to target parasympathetic fibres and inhibition of acetylcholine release. A pilot study of injection of 50 units of OnaB-A into the SPG was found to be safe, and further study is ongoing (NCT04069897) [78].

In a recent anatomical study, the location of the four temporal injections has been suggested to be altered in order to improve efficacy by better corresponding with the surface anatomy of the auriculotemporal nerve and awaits clinical confirmation [79]. Finally, an alternate strategy of injecting OnaB-A to the cranial sutures with ultrasound guidance has been suggested. The author supports this approach based on pre-clinical studies in which meningeal nociceptor sensitivity is reduced with this approach and the concentration of extracranial pain fibres in proximity to suture sites [80]. Clinical study is required to further evaluate this hypothesis.

### 7.2. Current Studies

There are several studies ongoing involving the use of botulinum toxin in both primary and secondary headache disorders. Selected trials that are currently registered on www.clinicaltrials.gov (accessed 3 May 2021) are presented in Table 2

## 8. Conclusions

The efficacy of onabotulinumtoxinA is clearly established as a treatment of chronic migraine. However, there remain several unanswered questions. Further work is required to assess prospectively markers of response and their inter-relationship, including iron deposition in the PAG and clinical duration of disease, unilateral pain, autonomic symptoms, allodynia and trigeminal activation and CGRP levels. Currently, there is insufficient evidence to accurately predict response to OnaB-A for an individual patient.

Further health-economic work is required to determine the optimal duration of a clinical trial of OnaB-A, with several studies suggesting the possibility of clinically meaningful response commencing after the second cycle. Considering the mechanism of action of OnaB-A, further studies optimising the PREEMPT protocol in an attempt to maximise its efficacy are also welcomed. 

## Figures and Tables

**Table 1:** Predictors of efficacy of onabotulinumtoxinA in chronic migraine.

Study	Population	Diagnosis	MOH	Therapy	Follow-Up (Months)	Definition of Response	Percentage Responders	Significant Predictors	Non-Significant Predictors
Eren, 2020 [32]	Germany, *n* = 49	100% CM	U	155 units OnaB-A PREEMPT	3	≥30% fewer MHD	38.8%	None	Triptan efficacy
Domínguez Vivero, 2020 [33]	Spain, *n* = 62	100% CM	U	155 units OnaB-A PREEMPT	3	≥50% fewer headaches	75.8%	Age, CGRP (>50 ng/mL), PTX3 (>1000 pg/mL), iron deposition in GP and PAG	Gender, BMI, smoking, phenotype (intensity, aura, duration), allodynia, presence or location of WML
Moreno-Mayordomo, 2019 [34]	Spain, *n* = 156	100% CM	U	155 units OnaB-A PREEMPT	6	≥50% fewer MMD	76.9%	Polymorphisms CALCA rs3781719 TRPV1 rs222749,	23 other SNPs
Schiano di Cola, 2019 [35]	Italy, *n* = 84	100% CM	65.5%	155–195 units OnaB-A PREEMPT	12	≥50% fewer headaches	73.9%	Depressive symptoms, MOH	N/A
de Tommaso, 2019 [36]	Italy, *n* = 120	100% CM	U	155–195 units OnaB-A PREEMPT	24	≥50% fewer headaches	61.6%	Less severe allodynia	N/A
Young, 2019 [37]	USA, Aus, Korea, *n* = 715	100% CM	U	155 units OnaB-A PREEMPT	27	Mean reduction MHD	N/A	absence allodynia (only at 27 months)	N/A
Domínguez, 2018 [38]	Spain, *n* = 62	100% CM	U	155 units OnaB-A PREEMPT	6	≥50% fewer headaches	75.8%	CGRP, PTX3	TNF-⍺, IL-10, IL-6, hs-CRP, cFn, S100, NSE
Domínguez, 2018 [39]	Spain, *n* = 725	100% CM	58.2%	155 units OnaB-A PREEMPT	12	≥50% fewer headaches	79.3%	CM duration, Unilateral pain, combined symptomatic treatment, fewer days of disability, milder headache	Gender, MA, depression, fibromyalgia,
Lovati, 2018 [40]	Italy, *n* = 44	100% CM	U	170–195 units OnaB-A PREEMPT	12	≥50% fewer MMD	31.8%	Triptan response	N/A
Hubbard, 2016 [41]	USA, *n* = 23	100% CM	U	150 units modif. OnaB-A PREEMPT	6	≥50% fewer MHD and now EM	47.8%	Cortical thickness at right SI and aINS, left ParsOp and STG	N/A
Lee, 2016 [42]	Korea, *n* = 70	100% CM	50% R, 47% NR	155 units OnaB-A PREEMPT	1.5	≥50% fewer MHD, ≥50%fewer abortive Rx OR ≥50% fewer mod-severe headache	60%	Disease duration MCA/ICA ratio	Age, gender, BMI, concurrent prophylactic medication, MA vs. MoA, psychiatric comorbidity, smoking
Cernuda-Morollón, 2015 [43]	Spain, *n* = 83	100% CM	31.3%	155–195 units OnaB-A PREEMPT	6	≥50% fewer headaches AND ≥50% reduced VAS	77.1%	CGRP (76.85 pg/mL vs. 50.45 pg/mL), VIP	Clinical features (MA), age, duration of CM, comorbidity, obesity, prophylactic medication
Lin, 2014 [44]	Taiwan, *n* = 94	100% CM	19.1%	75–155 units OnaB-A	3	≥30% fewer headaches	39.4%	Phenotype (ocular)	Phenotype (imploding vs. exploding), gender, MA, BMI, depression, dosage of OnaB-A
Pagola, 2014 [45]	Spain, *n* = 39	‘refractory migraine’	50% R, 81% NR	U	U	≥50% fewer MHD	46.2%	Nil	Unilateral location, implosive pain, pericranial muscle tension, duration of migraine, MOH
Bumb, 2013 [46]	Switzerland, *n* = 111	U	U	100 units OnaB-A	U	≥3 cycles of OnaB-A	42.3%	None	WML
Grogan, 2013 [47]	USA, *n* = 128	U, EM + CM	U	RimB-B	U	≥75% fewer headaches	79%	MA, phenotype (imploding, ocular)	Gender, dosage,
Kim, 2010 [48]	USA, *n* = 18	88.9% EM 11.1% CM	U	16–78 units OnaB-A	3	≥50% fewer headaches	72.2%	Phenotype (imploding pain)	N/A
Burstein, 2009 [49]	USA, *n* = 82	32.9% EM 67.1% CM	U	U	U	≥66.7% fewer MMD	45.1%	Phenotype of pain (imploding or ocular)	EM vs. CM
Mathew, 2008 [50]	USA, *n* = 71	100% CM	U	100 units OnaB-A	7	≥50% fewer headache AND ≥50% reduced MIDAS	76.1%	Unilateral headache, curaneous alloydrnia, pericranial muscle tenderness	N/A
Jakubowski, 2006 [51]	USA, *n* = 63	57.1% EM 42.9% CM	U	100 units OnaB-A	6	≥80% fewer MMD	61.9%	Phenotype of pain (imploding or ocular)	Neck muscles tenderness, EM vs. CM
Eross, 2005 [52]	USA, *n* = 74	23% EM 77% CM	23%	25–100 units OnaB-A	3	≥50% reduced disability	62%	Age, shorter duration of illness	EM vs. CM, MOH, dosage, muscle tenderness,

aINS—anterior insula, Aus—Australia, BMI—body mass index, cFn—cellular fibronectin, CGRP—calcitonin gene related peptide, CM—chronic migraine, EM—episodic migraine, GP—globus pallidus, hsCRP—high-sensitivity C-reactive protein, MA—migraine with aura, MCA/ICA ratio—middle cerebral artery, internal cerebral artery ratio (assessed by doppler ultrasound), MHD—monthly headache days, MIDAS—migraine disability assessment, MMD—monthly migraine days, MoA—migraine without aura, MOH—medication overuse headache, N/A—not applicable, NR—non-responder, NSE—neuron-specific enolase, OnaB-A—onabotulinumtoxinA, PAG—periaqueductal gray, ParsOp—pars opercularis, PREEMPT—Phase 3 research evaluation migraine prophylaxis therapy protocol for onabotulinumtoxinA injection, PTX3—pentraxin-related peptide, R—responder, RimB-B—rimabotulinumtoxinB, Rx—treatment, SI—Somatosensory cortex, SNPs—single nucleotide polymorphisms, STG—superior temporal gyrus, sTWEAK—tumour necrosis factor weak inducers of apoptosis in soluble form, TNF-⍺—tumour necrosis factor ⍺, U—unknown, USA—United states of America, VAS—visual analogue scale, WML—white matter lesion.

**Table 2:** Selected studies currently active involving botulinum toxin.

Brief Study Synopsis	Year Posted	Current Status	Trial Identifier
PraB-A for treatment of chronic migraine	2021	Recruiting	NCT04845178
OnaB-A for the treatment of post-stroke and vascular headache	2020	Not yet recruiting	NCT04580238
155 units vs. 100 units of OnaB-A for treatment of chronic migraine	2020	Recruiting	NCT04349176
OnaB-A blockade of the SPG for refractory chronic migraine	2019	Recruiting	NCT04069897

PraB-A—prabotulinumtoxinA, OnaB-A—onabotulinumtoxinA, SPG—sphenopalatine ganglia.
